# Continuous Expression of Interferon Regulatory Factor 4 Sustains CD8^+^ T Cell Immunity against Tumor

**DOI:** 10.34133/research.0271

**Published:** 2023-11-17

**Authors:** Anze Yu, Jinfei Fu, Zheng Yin, Hui Yan, Xiang Xiao, Dawei Zou, Xiaolong Zhang, Xiongbing Zu, Xian C. Li, Wenhao Chen

**Affiliations:** ^1^ Immunobiology and Transplant Science Center, Department of Surgery, Houston Methodist Research Institute and Institute for Academic Medicine, Houston Methodist Hospital, Houston, TX, USA.; ^2^ Department of Urology, First Affiliated Hospital, Sun Yat-sen University, Guangzhou, Guangdong, China.; ^3^ Systems Medicine and Bioengineering Department, Houston Methodist Neal Cancer Center, Houston, TX, USA.; ^4^ Department of Radiology, Houston Methodist Hospital, Weill Cornell Medicine, Houston, TX, USA.; ^5^ Department of Urology, Xiangya Hospital, Central South University, Changsha, Hunan, China.; ^6^ Department of Surgery, Weill Cornell Medicine, Cornell University, New York, NY, USA.

## Abstract

T-cell-based immunotherapy is gaining momentum in cancer treatment; however, our comprehension of the transcriptional regulation governing T cell antitumor activity remains constrained. The objective of this study was to explore the function of interferon regulatory factor 4 (IRF4) in antitumor CD8^+^ T cells using the TRAMP-C1 prostate cancer and B16F10 melanoma model. To achieve this, we generated an *Irf4*^GFP-DTR^ mouse strain and discovered that CD8^+^ tumor-infiltrating lymphocytes (TILs) expressing high levels of IRF4.GFP exhibited a more differentiated PD-1^high^ cell phenotype. By administering diphtheria toxin to tumor-bearing *Irf4*^GFP-DTR^ mice, we partially depleted IRF4.GFP^+^ TILs and observed an accelerated tumor growth. To specifically explore the function of IRF4 in antitumor CD8^+^ T cells, we conducted 3 adoptive cell therapy (ACT) models. Firstly, depleting IRF4.GFP^+^ CD8^+^ TILs derived from ACT significantly accelerated tumor growth, emphasizing their crucial role in controlling tumor progression. Secondly, deleting the *Irf4* gene in antitumor CD8^+^ T cells used for ACT led to a reduction in the frequency and effector differentiation of CD8^+^ TILs, completely abolishing the antitumor effects of ACT. Lastly, we performed a temporal deletion of the *Irf4* gene in antitumor CD8^+^ T cells during ACT, starting from 20 days after tumor implantation, which significantly compromised tumor control. Therefore, sustained expression of IRF4 is essential for maintaining CD8^+^ T cell immunity in the melanoma model, and these findings carry noteworthy implications for the advancement of more potent immunotherapies for solid tumors.

## Introduction

Cancer immunotherapy entails utilizing the body’s immune system to combat cancer cells, serving as a treatment approach [1–5]. The capability of T cells in combating cancer has been demonstrated through chimeric antigen receptor T-cell (CAR-T) therapy and immune checkpoint blockade (ICB), leading to the growing prominence and prevalence of T cell-based immunotherapy in cancer treatment [6,7]. However, the efficacy of T cells in attacking cancer is constrained by the suppressive tumor microenvironment (TME) and the progression of T cell exhaustion upon chronic antigen exposure [8–12]. Although current checkpoint blockade therapies have shown success in overcoming these limitations in some cancer patients, we now understand that the PD-1/PD-L1 checkpoint blockade primarily rejuvenates TCF1^+^ exhaustion precursor T cells, and using this approach alone does not prevent the commitment to the T cell exhaustion fate [13–16]. Most recently, a combined therapy involving PD-1 blockade plus IL-2 and a new immunocytokine PD1-IL2v have been shown to redirect the fate of TCF1^+^ antitumor T cells toward the effector cells [17,18]. Therefore, our understanding of T cell antitumor immunity remains evolving.

Transcription factors are crucial for precise control of gene expression, exerting a substantial impact on cell differentiation and enabling cells to carry out their specific functions. In the context of T cell exhaustion driven by persistent antigen/TCR stimulation, transcription factors downstream of the TCR-nuclear factor of activated T cells (NFAT) signaling pathway are considered key drivers of CD8^+^ T cell exhaustion. Among these T cell transcription factors, the thymocyte selection-associated high mobility group box protein (TOX) and the nuclear receptor 4A (NR4A) family have been identified [19–23]. The TCR-NFAT-TOX/NR4A axis holds a vital role in inducing CD8^+^ T cell exhaustion, facilitating the upregulation of various inhibitory receptors [19–23]. However, it is worth noting that TOX also contributes to the enduring presence of antigen-specific CD8^+^ T cells during chronic infections and in the context of cancer [20,21,24]. While eliminating NR4As has shown promise in improving T cell antitumor immunity in murine models [22], caution should be exercised when considering the manipulation of TOX. Therefore, the question arises as to whether targeting T cell exhaustion implicated transcription factors could be a viable strategy to enhance cancer immunotherapy.

We adopt a novel perspective in our investigation of transcriptional regulation in T cell antitumor immunity. Instead of focusing on transcription factors associated with T cell exhaustion, we propose studying the transcription factors responsible for sustaining effector function. Specifically, we emphasize the role of interferon regulatory factor 4 (IRF4), a transcription factor predominantly expressed in immune cells. In T cells, IRF4 instructs the differentiation of various T cell subsets, encompassing T helper (Th) 2, Th9, Th17, effector regulatory T cells, follicular helper T cells, and cytotoxic CD8^+^ T cells [25–30]. Experimental evidence has demonstrated that IRF4 deficiency compromises T cell immunity in microbial infections, allergies, autoimmunity, graft-versus-host reactions, and transplant rejection [27,29,31–33]. While IRF4 governs T cell effector function in different disease contexts, its role in antitumor immunity remains largely unexplored. Thus, we focused on examining the significance of IRF4 in antitumor CD8^+^ T cells, as they are pivotal in directly combating cancer. Through 3 distinct adoptive cell therapy (ACT) models, we discovered the essential role of IRF4-expressing CD8^+^ tumor-infiltrating lymphocytes (TILs) in murine melanoma defense. Moreover, our findings revealed that sustained expression of IRF4 is crucial for preserving CD8^+^ T cell immunity against murine melanoma. These insights will redefine strategic approaches to exploit transcriptional regulation for enhancing immunotherapies targeting solid tumors.

## Results

### IRF4 expression is positively correlated with a more differentiated phenotype of CD8^+^ T cells within melanoma

**Fig. 1.**
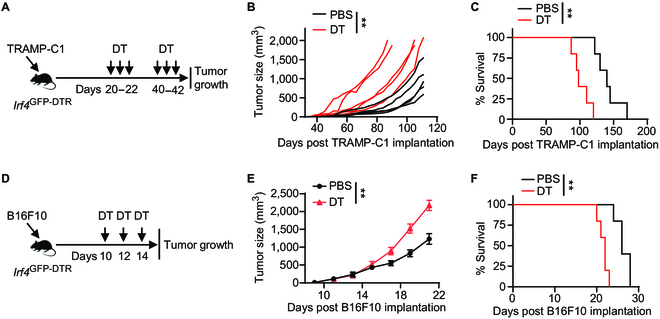
Effects of DT treatment on tumor growth in *Irf4*^GFP-DTR^ mice. (A to C) *Irf4* ^GFP-DTR^ mice were subcutaneously (s.c.) implanted with 2 × 10^6^ TRAMP-C1 prostate cancer cells and received treatment with 25 μg/kg DT or PBS vehicle control on the indicated days. (A) Experimental design depicting the timeline of treatments. (B and C) Tumor volumes and survival rates of TRAMP-C1 tumor-bearing mice in the DT and PBS treatment groups (*n* = 5 per group). (D to F) *Irf4* ^GFP-DTR^ mice were s.c. injected with 0.1 × 10^6^ B16F10 melanoma cells and treated with 25 μg/kg DT or PBS on the indicated days. (D) Experimental design illustrating the timing of treatments. (E and F) Mean tumor volumes and survival rates of B16F10 tumor-bearing mice in the DT and PBS treatment groups (*n* = 5 per group). In B, tumor growth curves were compared between the DT and PBS treatment groups using a 2-way ANOVA (mixed-effects model) with the Geisser–Greenhouse correction. In E, data are presented as mean ± SD, and tumor growth curves were compared between the DT and PBS treatment groups using a repeated measures 2-way ANOVA with the Geisser–Greenhouse correction. In C and F, survival rates were compared between the DT and PBS treatment groups using a log-rank test. **P < 0.01.

To examine the function of IRF4 in CD8^+^ T cell antitumor immunity, we generated *Irf4*^GFP-DTR^ mice using the CRISPR/Cas9 technique [34]. In these mice, a P2a.eGFP_P2a.DTR_stop cassette was inserted after the last exon of wild-type (WT) B6 mouse *Irf4*. The GFP knock-in serves as a reporter system for tracking IRF4 expression in immune cells, while the DTR knock-in allows for the depletion of IRF4-expressing cells in vivo (Fig. S1A). In this study, *Irf4*^GFP-DTR^ mice were subcutaneously (s.c.) implanted with 2 × 10^6^ TRAMP-C1 prostate cancer cells and treated with either 25 μg/kg body weight of diphtheria toxin (DT) or PBS vehicle on days 20, 21, 22, 40, 41, and 42 after tumor inoculation (Fig. 1A). Tumor growth was monitored, and the results revealed that TRAMP-C1 tumors in DT-treated *Irf4*^GFP-DTR^ mice exhibited faster growth compared to those in PBS-treated *Irf4*^GFP-DTR^ mice. This accelerated tumor growth led to significantly shortened animal survival (Fig. 1B and C). Additionally, we employed another model where *Irf4*^GFP-DTR^ mice were s.c. injected with 0.1 × 10^6^ B16F10 melanoma cells and treated with 25 μg/kg body weight of DT or PBS on days 10, 12, and 14 after tumor implantation (Fig. 1D). In this case, DT treatment significantly accelerated B16F10 tumor progression and shortened animal survival compared to the PBS injection (Fig. 1E and F).

**Fig. 2.**
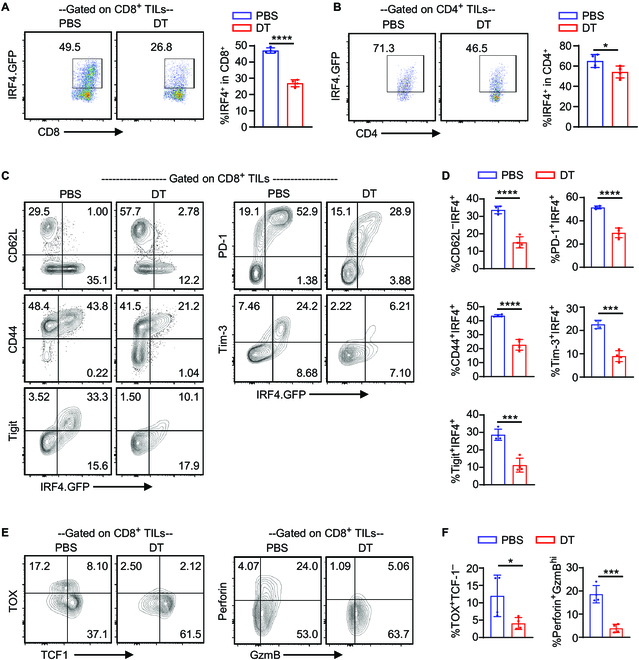
Differentiation phenotype of IRF4^+^ CD8^+^ TILs in melanoma. *Irf4* ^GFP-DTR^ mice were s.c. injected with 0.1 × 10^6^ B16F10 melanoma cells and treated with 25 μg/kg DT or PBS on days 10, 12, and 14 after tumor implantation. TILs were obtained on day 22 for flow cytometry analysis. (A and B) Representative flow cytometry plots and bar graphs showing the percentage of IRF4.GFP^+^ cells among CD8^+^ and CD4^+^ TILs in the PBS or DT treatment groups. (C and D) Representative flow cytometry plots and bar graphs illustrate the percentages of IRF4.GFP^+^CD62L^–^, IRF4.GFP^+^CD44^+^, IRF4.GFP^+^PD-1^+^, IRF4.GFP^+^Tim-3^+^, and IRF4.GFP^+^Tigit^+^ cells among CD8^+^ TILs in the PBS or DT treatment groups. (E and F) Percentages of TOX^+^TCF-1^–^ and Perforin^+^GzmB^high^ cells among CD8^+^ TILs in the PBS or DT treatment groups. In E, flow cytometry plots were gated on CD8^+^ TILs. The results in the bar graphs are shown as mean ± SD (*n* = 4). Statistical significance was determined using an unpaired 2-tailed Student’s *t* test. *P < 0.05, ***P < 0.001, ****P < 0.0001.

Since our study focuses on the role of IRF4 in antitumor CD8^+^ T cells, we conducted a further analysis of TILs in *Irf4*^GFP-DTR^ mice with B16F10 tumors. On day 22 after B16F10 implantation, TILs were examined using flow cytometry in both the PBS- and DT-treated groups. The gating strategy for detecting CD8^+^ and CD4^+^ TILs in B16F10 tumors is illustrated in Fig. S1B. Notably, DT treatment significantly decreased the frequencies of IRF4.GFP^+^ cells in both CD8^+^ and CD4^+^ TILs (Fig. 2A and B).

The expression of IRF4.GFP in CD8^+^ TILs showed a strong correlation with markers associated with a more differentiated T cell phenotype, such as PD-1, Tim-3, Tigit, CD44^+^, and CD62L^–^ (Fig. 2C and D). In the DT-treated group, there was a significant decrease in the frequencies of PD-1^+^IRF4.GFP^+^, Tim3^+^IRF4.GFP^+^, and Tigit^+^IRF4.GFP^+^ cells within the CD8^+^ TIL population (Fig. 2C and D). Additionally, DT treatment led to a notable reduction in the frequencies of TOX^+^TCF1^–^ and Perforin^+^Granzyme B^hi^ cells within the CD8^+^ TIL population (Fig. 2E and F). Within the CD4^+^ TIL population, the frequencies of CD62L^–^CD44^+^, Tim3^+^IRF4.GFP^+^, and Tigit^+^IRF4.GFP^+^ cells were also significantly decreased in the DT-treated group (Fig. S1C and D). These findings collectively indicate that IRF4.GFP^+^ TILs exhibit a more differentiated T cell phenotype. Moreover, impaired melanoma control in the DT-treated group is associated with the partial depletion of IRF4.GFP^+^ T cells within the tumor.

### Adoptively transferred antitumor CD8^+^ T cells exhibit high expression of IRF4 within the melanoma

The effectiveness of the immune response against tumors greatly relies on the crucial function of CD8^+^ T cells. To explore the potential role of IRF4 in regulating their antitumor activities, we generated *Irf4*^GFP-DTR^ Thy1.1^+^ Pmel-1 mice. The TCR transgenic Pmel-1 CD8^+^ T cells recognize melanoma-melanocyte antigen gp100. After being stimulated in vitro with the hgp100_25-33_ peptide, Pmel-1 CD8^+^ T cells derived from *Irf4*^GFP-DTR^ Thy1.1^+^ Pmel-1 mice became activated and exhibited IRF4.GFP expression (Fig. S2A).

**Fig. 3.**
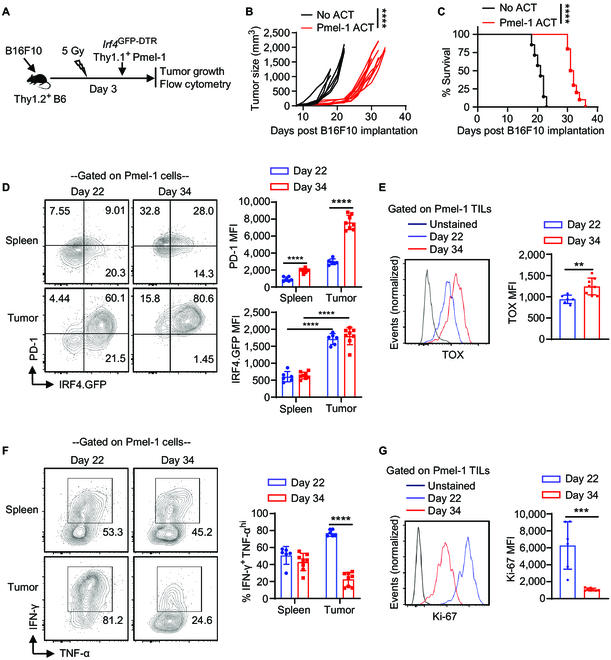
IRF4 expression in adoptively transferred antitumor CD8^+^ T cells in melanoma. Thy1.2^+^ B6 mice were s.c. injected with 0.2 × 10^6^ B16F10 cells on day 0. The mice were then sub-lethally irradiated and adoptively transferred with 2 × 10^6^ activated *Irf4*^GFP-DTR^Thy1.1^+^ Pmel-1 CD8^+^ T cells (Pmel-1 ACT) or without any T cell transfer (No ACT) on day 3. Tumor growth was monitored, and adoptively transferred Pmel-1 cells were analyzed on days 22 and 34. (A) Schematic of the experimental design. (B and C) Tumor volumes and survival rates of B16F10 tumor-bearing mice in the Pmel-1 ACT (*n* = 10) and No ACT (*n* = 7) groups. (D) IRF4.GFP and PD-1 expression of Pmel-1 cells in spleens and tumors of the Pmel-1 ACT group on the indicated days. (E) TOX expression of Pmel-1 TILs on indicated days. (F) Percentage of IFN-γ^+^TNF-α^high^ cells among Pmel-1 T cells in spleens and tumors. (G) Ki-67 expression of Pmel-1 TILs. In B, tumor growth curves (from day 8 to day 22) were compared between the Pmel-1 ACT and No ACT groups using a 2-way ANOVA (mixed-effects model) with the Geisser–Greenhouse correction. In C, survival rates were compared between the Pmel-1 ACT and No ACT groups using a log-rank test. In D to G, the results in bar graphs are presented as mean ± SD (*n* = 6 to 8), and statistical significance was determined using an unpaired 2-tailed Student’s *t* test. **P < 0.01, ***P < 0.001, ****P < 0.0001.

The ACT model using Pmel-1 cells is a suitable approach for studying CD8^+^ T cell immunity in murine melanoma. To establish this model, Thy1.2^+^ B6 mice received a subcutaneous injection of 0.2 × 10^6^ B16F10 cells. On day 3 after tumor implantation, the mice underwent sub-lethal irradiation. Within 6 h after irradiation, the mice were either adoptively transferred with 2 × 10^6^ activated *Irf4*^GFP-DTR^ Thy1.1^+^ Pmel-1 CD8^+^ T cells (Pmel-1 ACT group) or were left without cell transfer (No ACT group) (Fig. 3A). Notably, compared to the No ACT group, Pmel-1 ACT exhibited inhibitory effects on B16F10 tumor growth and significantly prolonged animal survival (Fig. 3B and C).

Flow cytometry analysis was performed on adoptively transferred *Irf4*^GFP-DTR^ Thy1.1^+^ Pmel-1 CD8^+^ T cells derived from spleens and tumors on days 22 and 34 after B16F10 implantation. The gating strategy for detecting Thy1.1^+^ Pmel-1 CD8^+^ T cells in B16F10 tumors is depicted in Fig. S3B. Remarkably, Pmel-1 cells within the tumors demonstrated notably elevated levels of IRF4.GFP expression compared to those in the spleens (Fig. 3D). Notably, between day 22 and day 34, Pmel-1 TILs underwent further phenotypic changes, characterized by increased expression of PD-1 and TOX (Fig. 3D and E), as well as a decrease in interferon (IFN)-γ production and Ki67 expression (Fig. 3F and G). These findings collectively underscore the elevated expression of IRF4 in adoptively transferred Pmel-1 cells within the melanoma.

### IRF4^+^ CD8^+^ TILs derived from ACT play an essential role in melanoma control

**Fig. 4.**
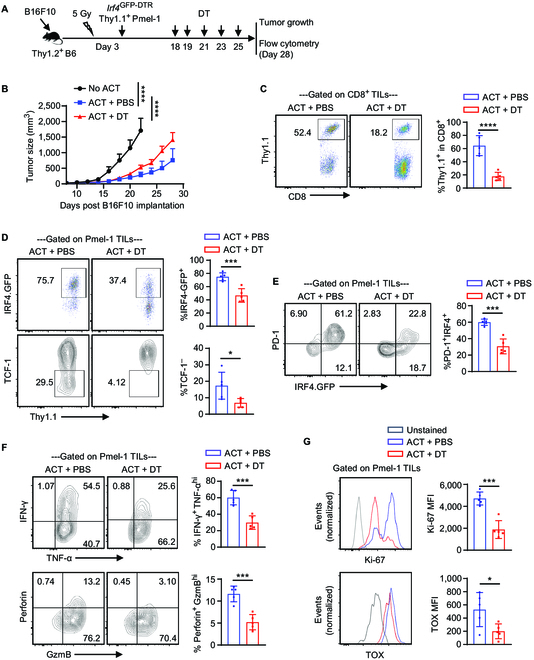
Effects of depleting IRF4^+^ CD8^+^ TILs derived from ACT on melanoma control. Thy1.2^+^ B6 mice were s.c. injected with 0.2 × 10^6^ B16F10 cells on day 0. The mice were sub-lethally irradiated and adoptively transferred with 2 × 10^6^ activated *Irf4*^GFP-DTR^Thy1.1^+^ Pmel-1 CD8^+^ T cells (ACT groups) or without any T cell transfer (No ACT group) on day 3. The mice in the ACT groups were further treated with 50 μg/kg DT (ACT + DT) or PBS (ACT + PBS) on indicated days. Tumor growth was monitored, and adoptively transferred Pmel-1 cells were analyzed on day 28. (A) Experimental design depicting the timeline of events. (B) Line graph showing mean ± SD tumor volumes of the indicated groups (*n* = 10 per group). (C) Percentage of Thy1.1^+^ Pmel-1 cells among CD8^+^ TILs in the indicated groups. (D to F) Percentages of IRF4.GFP^+^ cells, TCF-1^–^ cells, PD-1^+^IRF4.GFP^+^ cells, IFN-γ^+^TNF-α^hi^ cells, and Perforin^+^GzmB^hi^ cells among Pmel-1 TILs in the indicated groups. (G) Ki-67 and TOX expression of Pmel-1 TILs in the indicated groups. In B, tumor growth curves were compared (No ACT vs. ACT + PBS, day 8 to day 22; ACT + DT vs. ACT + PBS) using a repeated measures 2-way ANOVA with the Geisser–Greenhouse correction. In D to F, data in bar graphs are presented as mean ± SD (*n* = 5), and statistical significance was determined using an unpaired 2-tailed Student’s *t* test. *P < 0.05, ***P < 0.001, and ****P < 0.0001.

To explore the influence of IRF4^+^ Pmel-1 cells in tumor control during ACT, Thy1.2^+^ B6 mice were subjected to subcutaneous injection of 0.2 × 10^6^ B16F10 cells. On day 3 after tumor implantation, the mice underwent sub-lethal irradiation and were subsequently adoptively transferred with 2 × 10^6^ activated *Irf4*^GFP-DTR^ Thy1.1^+^ Pmel-1 CD8^+^ T cells (ACT groups) or left without cell transfer (No ACT group). The ACT groups received additional treatment with 50 μg/kg DT (ACT + DT group) or PBS (ACT + PBS group) on days 18, 19, 21, 23, and 25 (Fig. 4A). Prior to DT administration, ACT of *Irf4*^GFP-DTR^ Thy1.1^+^ Pmel-1 CD8^+^ T cells significantly inhibited tumor growth compared to the No ACT group. However, following DT or PBS treatment, tumor growth was significantly accelerated in the ACT + DT group compared to the ACT + PBS group (Fig. 4B).

To investigate the impact of DT administration on the efficacy of Pmel-1 ACT, the *Irf4*^GFP-DTR^ Thy1.1^+^ Pmel-1 TILs derived from ACT were analyzed on day 28 after B16F10 implantation. Notably, DT administration resulted in a significant reduction in the proportion of Pmel-1 TILs within the total CD8^+^ TIL population (Fig. 4C). Furthermore, DT treatment led to a significant decrease in the frequencies of IRF4.GFP^+^ cells among the remaining Pmel-1 TILs (Fig. 4D). Intriguingly, DT administration also resulted in the depletion of terminally differentiated TCF1^–^ Pmel-1 TILs (Fig. 4D).

Among Pmel-1 TILs, IRF4.GFP^+^ cells displayed high levels of PD-1 expression, while IRF4.GFP^–^ cells did not. DT administration primarily depleted the IRF4.GFP^+^PD-1^+^ Pmel-1 TILs (Fig. 4E) and reduced the frequency of IFN-γ and perforin-producing cells (Fig. 4F). Additionally, remaining Pmel-1 TILs in the DT-treated group exhibited lower levels of TOX expression and the proliferation marker Ki67 compared to those in the PBS-treated group (Fig. 4G). Taken together, IRF4.GFP^+^ Pmel-1 TILs derived from ACT play an essential role in melanoma control.

### The deletion of the *Irf4* gene in antitumor CD8^+^ T cells eliminates the antitumor effects of ACT

Considering that a majority of CD8^+^ TILs derived from ACT exhibit an IRF4^+^ cell phenotype, we aimed to examine the involvement of IRF4 in antitumor CD8^+^ T cells. Effector differentiation in CD8^+^ T cells is often associated with the loss of TCF1 expression [35,36]. To assess the effects of IRF4 deletion on effector differentiation, we generated TCF1^GFP^ Thy1.1^+^ Pmel-1 and *Irf4*^–/–^TCF1^GFP^ Thy1.1^+^ Pmel-1 mice. Following in vitro stimulation with hgp100_25-33_ stimulation, *Irf4*^–/–^TCF1^GFP^ Pmel-1 cells exhibited no IRF4 expression but maintained high levels of TCF1.GFP expression. In contrast, TCF1^GFP^ Pmel-1 cells displayed elevated IRF4 expression and downregulated TCF1.GFP expression (Fig. S3).

**Fig. 5.**
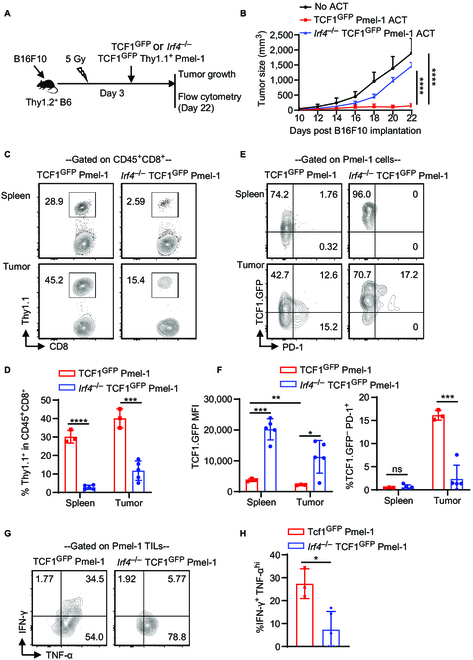
Effects of deleting *Irf4* gene in CD8^+^ T cells of ACT on their antitumor activity. Thy1.2^+^ B6 mice were s.c. injected with 0.2 × 10^6^ B16F10 cells on day 0. On day 3, the mice were sub-lethally irradiated and either left without any T cell transfer (No ACT) or adoptively transferred with 2 × 10^6^ activated TCF1^GFP^ Thy1.1^+^ Pmel-1 or *Irf4^–/–^* TCF1^GFP^ Thy1.1^+^ Pmel-1 CD8^+^ T cells. Tumor growth was monitored, and adoptively transferred Pmel-1 cells were analyzed on day 22. (A) Schematic of the experimental design. (B) Mean tumor volumes of the indicated groups (mean ± SD, *n* = 5 per group). (C and D) Percentage of Thy1.1^+^ Pmel-1 cells within CD8^+^ T cells in spleens and tumors of the TCF1^GFP^ Thy1.1^+^ Pmel-1 or *Irf4^–/–^*TCF1^GFP^ Thy1.1^+^ Pmel-1 ACT groups. (E and F) Percentage of TCF1.GFP^–^ PD-1^+^ cells and TCF1.GFP MFI of Pmel-1 CD8^+^ T cells in spleens and tumors of indicated ACT groups. (G and H) Percentage of IFN-γ^+^TNF-α^hi^ cells among Pmel-1 TILs in the indicated ACT groups. In B, tumor growth curves were compared between indicated groups using a repeated measures 2-way ANOVA with the Geisser–Greenhouse correction. In D, F, and H, the results in bar graphs are presented as mean ± SD (*n* = 3 to 5), and statistical significance was determined using an unpaired 2-tailed Student’s *t* test. ns, *P* > 0.05; *P < 0.05, **P < 0.01, ***P < 0.001, ****P < 0.0001.

Next, Thy1.2^+^ B6 mice were subjected to subcutaneous injection of 0.2 × 10^6^ B16F10 cells and sub-lethally irradiated on day 3 after tumor implantation. Six hours after irradiation, the mice were either left without cell transfer (No ACT group) or adoptively transferred with 2 × 10^6^ activated TCF1^GFP^ Thy1.1^+^ Pmel-1 or *Irf4*^–/–^TCF1^GFP^ Thy1.1^+^ Pmel-1 cells (Fig. 5A). TCF1^GFP^ Pmel-1 ACT significantly inhibited B16F10 tumor growth, while tumor growth in the *Irf4*^–/–^TCF1^GFP^ Pmel-1 ACT group was comparable to the No ACT group (Fig. 5B). Therefore, the deletion of IRF4 in antitumor CD8^+^ T cells eliminates the efficacy of ACT.

Splenocytes and TILs were collected from the ACT groups on day 22 after tumor implantation. The frequencies of adoptively transferred *Irf4*^–/–^TCF1^GFP^ Pmel-1 cells in spleens and tumors were significantly lower compared to TCF1^GFP^ Pmel-1 cells (Fig. 5C and D). In both spleens and tumors, *Irf4*^–/–^TCF1^GFP^ Pmel-1 cells largely retained TCF1.GFP expression. Within the B16F10 tumors, TCF1^GFP^ Pmel-1 TILs, but not *Irf4*^–/–^TCF1^GFP^ Pmel-1 TILs, demonstrated the potential to develop into TCF1^–^PD-1^+^ cells (Fig. 5E and F). Moreover, the percentage of IFN-γ-producing cells among *Irf4*^–/–^TCF1^GFP^ Pmel-1 TILs was significantly lower than that among TCF1^GFP^ Pmel-1 TILs (Fig. 5G and H). Collectively, deleting the *Irf4* gene in antitumor CD8^+^ T cells abolishes the antitumor effects of ACT.

### The temporal deletion of *Irf4* in antitumor CD8^+^ T cells following ACT impairs tumor control

**Fig. 6.**
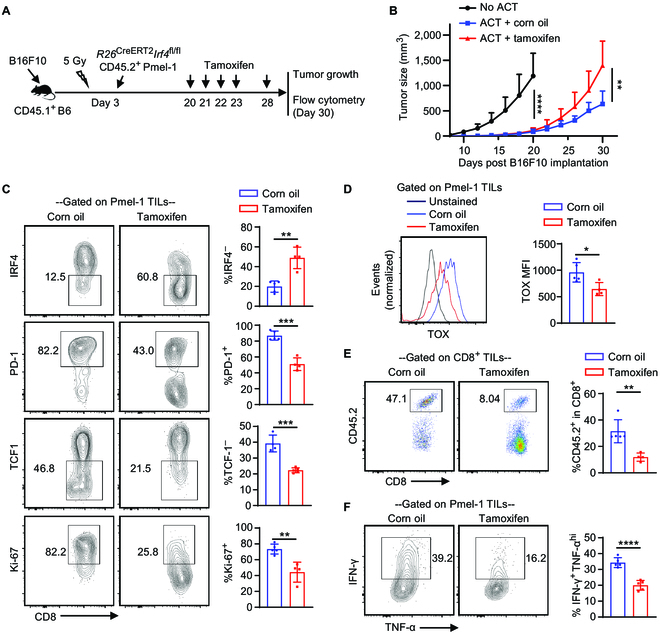
Effects of deleting the *Irf4* gene in antitumor CD8^+^ T cells after ACT on melanoma control. CD45.1^+^ B6 mice were s.c. injected with 0.2 × 10^6^ B16F10 cells on day 0. On day 3, the mice were sub-lethally irradiated and adoptively transferred with (ACT groups) or without (No ACT) 2 × 10^6^ activated *R26*^CreERT2^*Irf4*^fl/fl^ CD45.2^+^ Pmel-1 cells. The mice in the ACT groups were further treated with 2 mg of tamoxifen (ACT + tamoxifen) or corn oil (ACT + corn oil) on indicated days. Tumor growth was monitored, and TILs were obtained on day 30 for flow cytometry analysis. (A) Experimental design illustrating the timeline of events. (B) Mean tumor volumes of the indicated groups (mean ± SD, *n* = 10 per group). (C) Percentages of IRF4^–^, PD-1^+^, TCF1^–^, and Ki67^+^ cells among CD8^+^CD45.2^+^ Pmel-1 TILs in corn oil- and tamoxifen-treated ACT groups. (D) TOX expression of Pmel-1 TILs in corn oil- and tamoxifen-treated ACT groups. (E) Percentage of CD45.2^+^ Pmel-1 cells among total CD8^+^ TILs in indicated treatment groups. (F) Percentage of IFN-γ^+^TNF-α^hi^ cells among Pmel-1 TILs in indicated treatment groups. In B, tumor growth curves were compared (No ACT vs. ACT + corn oil, day 8 to day 20; ACT + corn oil vs. ACT + tamoxifen) using a repeated measures 2-way ANOVA with the Geisser–Greenhouse correction. In C to F, data in bar graphs are presented as mean ± SD (*n* = 4 to 5), and statistical significance was determined using an unpaired 2-tailed Student’s *t* test. *P < 0.05, **P < 0.01, ***P < 0.001, and ****P < 0.0001.

To further elucidate the role of IRF4 in antitumor CD8^+^ T cells, we generated *R26*^CreERT2^*Irf4*^fl/fl^ CD45.2^+^ Pmel-1 mice. Briefly, CD45.1^+^ B6 mice were subjected to subcutaneous injection of 0.2 × 10^6^ B16F10 cells. On day 3 after tumor implantation, the mice underwent sub-lethal irradiation and were subsequently adoptively transferred with 2 × 10^6^ activated *R26*^CreERT2^*Irf4*^fl/fl^ CD45.2^+^ Pmel-1 cells (ACT groups) or remained without cell transfer (No ACT group). The ACT groups received further treatment with either 2 mg of tamoxifen (ACT + tamoxifen) or corn oil vehicle (ACT + corn oil) on days 20, 21, 22, 23, and 28 after tumor implantations (Fig. 6A). Prior to tamoxifen or corn oil administration, ACT of *R26*^CreERT2^*Irf4*^fl/fl^ CD45.2^+^ Pmel-1 cells significantly suppressed tumor growth compared to the No ACT group. However, upon tamoxifen or corn oil treatment, tumor growth was notably accelerated in the ACT + tamoxifen group in comparison to the ACT + corn oil group (Fig. 6B).

To investigate the effects of tamoxifen on the adoptively transferred *R26*^CreERT2^*Irf4*^fl/fl^ CD45.2^+^ Pmel-1 cells, TILs were obtained from the ACT groups on day 30 after tumor implantation and subjected to flow cytometry analysis. Tamoxifen administration significantly abrogated IRF4 expression in the majority of Pmel-1 TILs (Fig. 6C, top panels). Compared to corn oil treatment, tamoxifen treatment substantially reduced the frequencies of PD-1^+^, TCF1^–^, and Ki67^+^ cells among Pmel-1 TILs (Fig. 6C) and decreased TOX expression in Pmel-1 TILs (Fig. 6D). Furthermore, tamoxifen treatment also significantly decreased the frequency of Pmel-1 cells among CD8^+^ TILs (Fig. 6E) and impaired the production of IFN-γ by Pmel-1 TILs (Fig. 6F). Collectively, the deletion of IRF4 in Pmel-1 ACT, starting at 20 days after tumor implantation, markedly impairs tumor control, which correlates with reduced frequency and function of Pmel-1 TILs.

## Discussion

The exploration of transcriptional regulation underlying CD8^+^ T cell immunity in solid tumors has been insufficient. In this study, we made a notable discovery that IRF4^+^ CD8^+^ TILs exhibited a more differentiated cell phenotype in melanoma-bearing *Irf4*^GFP-DTR^ mice. Accelerated tumor growth was found upon DT treatment, which correlated with the depletion of IRF4^+^ TILs. However, considering that IRF4 can be expressed in various immune cell types, DT treatment may impact multiple immune cell subsets in this model. Hence, to specifically explore the function of IRF4 in antitumor CD8^+^ T cells, we developed 3 distinct Pmel-1 ACT models: one involving the in vivo depletion of IRF4^+^ TILs derived from ACT, another with the deletion of the *Irf4* gene in antitumor CD8^+^ T cells used for ACT, and a third with the temporal deletion of the *Irf4* gene in antitumor CD8^+^ T cells following ACT. Through these complementary approaches, we demonstrated that IRF4^+^ TILs play a crucial role in tumor control. Furthermore, our findings highlight the significance of sustained IRF4 expression for maintaining CD8^+^ T cell immunity against melanoma.

Recent studies have shed light on the presence of exhausted CD8^+^ TIL populations in different types of human cancers. These CD8^+^ TILs are characterized by the expression of genes such as *TOX*, *PDCD1*, and *ENTPD1*, while still retaining the expression of effector genes *IFNG* and *GZMB* [37,38]. The exact antitumor function of these CD8^+^ TILs remains poorly understood and necessitates further investigation. Our research in melanoma-bearing *Irf4*^GFP-DTR^ mice revealed a major subset of endogenous IRF4.GFP^+^ CD8^+^ TILs exhibiting high levels of PD-1, Tim-3, and TIGIT. Although the expression of these inhibitory receptors is often linked to CD8^+^ T cell exhaustion, it is crucial to recognize that PD-1, Tim-3, and TIGIT are also T cell activation markers. Following DT treatment, a decline in tumor control correlated with the depletion of PD-1^+^IRF4.GFP^+^, Tim-3^+^IRF4.GFP^+^, and TIGIT^+^IRF4.GFP^+^ CD8^+^ TILs, indicating that these markers denote activated and functional TILs in this context. Regardless of interpretation, TILs expressing these receptors undeniably contribute to antitumor immunity.

In our study utilizing the *Irf4*^GFP-DTR^ Pmel-1 ACT model, we investigated further into the functionality of antitumor CD8^+^ T cells. While the adoptive transfer of *Irf4*^GFP-DTR^ Pmel-1 cells effectively suppresses tumor growth following melanoma implantation, an inevitable progression of these tumors is observed in later stages. A potential explanation for this could be the requirement for a diverse TCR repertoire among CD8^+^ TILs to ensure a robust antitumor response. Given that Pmel-1 cells have a single transgenic TCR targeting a specific tumor-associated antigen, their potential to fully eradicate the implanted melanoma may be limited. However, it is important to note that when IRF4.GFP^+^ Pmel-1 TILs were depleted, there was a significant decrease in the efficacy of the ACT therapy, underscoring the crucial role these cells play in antitumor immunity.

The critical role of IRF4 in T cell effector function has been extensively demonstrated across various T cell effector subsets and models of T cell-mediated diseases [25–33]. For instance, in transplantation models, our previous research clearly established that the absence of IRF4 leads to CD4^+^ T cell dysfunction and completely abolishes CD8^+^ T cell effector differentiation [33,39,40]. In this study, we observed that IRF4-deficient antitumor CD8^+^ T cells displayed no discernible antitumor effects in the ACT model. More importantly, when we temporally deleted the *Irf4* gene in antitumor CD8^+^ T cells after ACT, starting at 20 days after melanoma implantation, we observed compromised tumor control. These findings unequivocally demonstrate that IRF4 serves as a transcriptional determinant crucial for the development of CD8^+^ T cell immunity against murine melanoma.

It is becoming increasingly evident that the PD-1/PD-L1 checkpoint blockade primarily enhances the activity of TCF1^+^ “progenitor-like” T cells [13–16]. An effective antitumor immune response may require the combined action of both TCF1^–^ effector cells and TCF1^+^ progenitor-like T cells. While effector cells possess the capability to kill tumor cells, progenitor-like T cells may both sustain themselves within tumors and replenish the effector cell pool. In our study that employed the *Irf4*^–/–^TCF1^GFP^ Pmel-1 ACT model, we observed that IRF4 deletion significantly reduced the frequency of transferred Pmel-1 cells in tumor-bearing mice. Notably, the remaining *Irf4*^–/–^ Pmel-1 cells predominantly exhibited TCF1.GFP expression. This suggests that IRF4 deletion not only reduces the expansion of antitumor T cells but also likely impedes the potential of TCF1^+^ progenitor-like T cells to differentiate into TCF1^–^ effector cells.

One limitation of our study is its exclusive focus on the transcriptional regulation of CD8^+^ T cell immunity in animal models. Further investigations are necessary to elucidate the molecular mechanisms underlying T cell function in human cancers. Additionally, since tumors contain abundant cognate antigens for infiltrating T cells, understanding the mechanisms of T cell exhaustion remains a crucial area of research. However, our present study primarily centers around the role of IRF4, a crucial transcription factor that plays a vital role in supporting and sustaining the function of antitumor CD8^+^ T cells. These findings have significant implications for advancing more potent immunotherapies that target solid tumors.

## Materials and Methods

### Mice

C57BL/6 (B6), CD45.1^+^ B6, *R26*^CreERT2^, *Irf4*^flox/flox^, Thy1.1^+^ Pmel-1 TCR-transgenic, and *Tcf7*^GFP^ flox (referred to as TCF1^GFP^) mice were acquired from the Jackson Laboratory (Bar Harbor, ME). *Irf4*^−/−^ mice have been documented in prior studies [33]. We designed the *Irf4*^GFP-DTR^ mice and enlisted the assistance of Jackson Laboratory Model Generation Services to create this mouse line through the utilization of the CRISPR/Cas9 methodology [34]. We conducted a cross between *Irf4*^GFP-DTR^ and Thy1.1^+^ Pmel-1 mice to generate *Irf4*^GFP-DTR^ Thy1.1^+^ Pmel-1 mice. *R26*^CreERT2^ mice were crossed to *Irf4*^flox/flox^ mice and then to Pmel-1 mice to generate *R26*^CreERT2^*Irf4*^fl/fl^ CD45.2^+^ Pmel-1 mice. TCF1^GFP^ and Thy1.1^+^ Pmel-1 mice were crossed to generate TCF1^GFP^ Thy1.1^+^ Pmel-1 mice. *Irf4*^−/−^ and TCF1^GFP^ Thy1.1^+^ Pmel-1 mice were crossed to generate *Irf4*^−/−^TCF1^GFP^ Thy1.1^+^ Pmel-1 mice. The Institutional Animal Care and Use Committee (IACUC) at Houston Methodist Research Institute granted approval for all animal-related procedures conducted in this study.

### Tumor cell line and cell culture

The B16F10 and TRAMP-C1 cell lines were obtained from the American Type Culture Collection (Manassas, VA). All cell lines were tested negative for mycoplasma and other pathogens with IDEXX BioAnalytics (Columbia, MO). B16F10 cells and TRAMP-C1 cells were cultured in Dulbecco’s Modified Eagle Medium supplemented with 10% heat inactivated FBS.

### In vivo tumor growth

For tumor growth experiments, subcutaneous injections were administered with 0.1 × 10^6^ B16F10 cells or 2 × 10^6^ TRAMP-C1 cells into the right flanks of *Irf4*^GFP-DTR^ mice, or 0.2 × 10^6^ B16F10 cells into the right flanks of WT B6 or CD45.1^+^ B6 mice.

The dimensions of the tumors were measured using a caliper every other day in 2 directions (length and width). The tumor volume was then computed using the following formula: volume (mm^3^) = (length × width^2^)/2. Tumor-bearing mice were allocated at random to different treatment groups, as detailed below. In terms of survival investigations, euthanasia was carried out either upon reaching the endpoint tumor volume (2,000 mm^3^) or diameter (20 mm), or when the mice displayed distress signals, following the guidelines outlined by IACUC.

### Depletion of IRF4.GFP^+^ T cells in tumor-bearing *Irf4*^GFP-DTR^ mice

DT (D0564, Sigma-Aldrich) was dissolved in PBS and used to deplete IRF4-expressing T cells in tumor-bearing *Irf4*^GFP-DTR^ mice. In the TRAMP-C1 implantation model, *Irf4*^GFP-DTR^ mice were s.c. injected with 2 × 10^6^ TRAMP-C1 prostate cancer cells on day 0 and intraperitoneally injected with 25 μg/kg of body weight DT or 100 μl of PBS vehicle on days 20, 21, 22, 40, 41, and 42. In the B16F10 implantation model, *Irf4*^GFP-DTR^ mice were injected with 0.1 × 10^6^ B16F10 cells on day 0 and intraperitoneally injected with 25 μg/kg DT or 100 μl of PBS vehicle on days 10, 12, and 14. Tumor growth curve and survival rate were determined. In the B16F10 model, the effects of DT administration on TILs were examined through flow cytometry analysis on day 22 after tumor implantation.

### In vitro activation of Pmel-1 T cells prior to ACT

Pmel-1 CD8^+^ T cells from *Irf4*^GFP-DTR^ Thy1.1^+^ Pmel-1, *R26*^CreERT2^*Irf4*^fl/fl^ CD45.2^+^ Pmel-1, TCF1^GFP^ Thy1.1^+^ Pmel-1, and *Irf4*^−/−^TCF1^GFP^ Thy1.1^+^ Pmel-1 mice were activated in vitro prior to Pmel-1 ACT. In brief, after lysing red blood cells with ACK lysis buffer (Thermo Fisher), splenocytes from the above mouse strains were stimulated with 1 μM hgp100_25-33_ peptide (GenScript) and 10 IU/ml recombinant human IL-2 (200-02, PeproTech) in complete RPMI 1640 medium. Forty-eight hours after peptide stimulation, activation status and frequency of Pmel-1 CD8^+^ T cells were verified by flow cytometry analysis. Cultured splenocytes containing 2 × 10^6^ activated Pmel-1 CD8^+^ T cells were transferred into B16F10 tumor-bearing WT B6 or CD45.1^+^ B6 mice.

### Pmel-1 ACT models

To examine the function of IRF4.GFP^+^ Pmel-1 cells in Pmel-1 ACT therapy, WT B6 (Thy1.2^+^) mice were s.c. injected with 0.2 × 10^6^ B16F10 cells on day 0. The mice were then sub-lethally irradiated (5 Gy) and adoptively transferred with (ACT groups) or without (No ACT group) 2 × 10^6^ activated *Irf4*^GFP-DTR^ Thy1.1^+^ Pmel-1 CD8^+^ T cells on day 3. The mice in the ACT groups were treated with 50 μg/kg DT on indicated days to deplete IRF4.GFP^+^ Pmel-1 T cells or treated with PBS as controls.

To define the role of IRF4 in the generation of exhaustion-like Pmel-1 TILs, Thy1.2^+^ WT B6 mice were s.c. injected with 0.2 × 10^6^ B16F10 cells. On day 3 after tumor cell implantation, the mice were sub-lethally irradiated and transferred with 2 × 10^6^ activated *Irf4*^–/–^TCF1^GFP^ Thy1.1^+^ Pmel-1 or TCF1^GFP^ Thy1.1^+^ Pmel-1 T cells. The mice that did not receive Pmel-1 cell transfer were served as No ACT controls.

To define the role of IRF4 in regulating the exhaustion-like Pmel-1 TILs, CD45.1^+^ B6 mice were s.c. injected with 0.2 × 10^6^ B16F10 cells on day 0. The mice were then sub-lethally irradiated and adoptively transferred with (ACT groups) or without (No ACT group) 2 × 10^6^ activated *R26*^CreERT2^*Irf4*^fl/fl^ CD45.2^+^ Pmel-1 T cells on day 3. The ACT groups were intraperitoneally injected with 2 mg of tamoxifen (T5648, Sigma-Aldrich) on indicated days to delete IRF4 in Pmel-1 cells or treated with corn oil as controls.

Following Pmel-1 ACT, tumor growth was monitored, and the adoptively transferred Pmel-1 T cells were analyzed by flow cytometry on indicated days.

### Tumor processing to analyze TILs

Mice harboring B16F10 tumors were humanely euthanized at specified time intervals. The tumors were excised, cut into small fragments, and subjected to enzymatic digestion at 37 °C for 30 min. The enzymatically treated tumor tissue was then mashed through a 70-μm cell strainer to yield single-cell suspensions. TILs were extracted from these single-cell suspensions through density gradient centrifugation using Ficoll (GE Healthcare). Following isolation, the cells were rinsed with PBS before undergoing flow cytometry analysis.

### Flow cytometry analysis

TILs and splenocytes obtained from the mice were treated with Zombie Aqua dye (BioLegend) for 15 min at room temperature to discriminate viable cells. For cell surface marker staining, fluorochrome-conjugated antibodies specific for murine CD4 (clone GK1.5), CD8 (53-6.7), CD45.1 (A20), CD45.2 (104), CD62L (MEL-14), CD44 (IM7), Tim-3 (B8.2C12), PD-1 (29F.1A12), Tigit (1G9), CD90.1 (OX-7), CD45 (30-F11), and TCR Vβ13 (MR12-4) were purchased from BioLegend.

Intracellular transcription factor staining was conducted using the Foxp3/Transcription Factor Staining Buffer Set (Thermo Fisher). Mouse-specific Ki-67 (16A8) was purchased from BioLegend, fluorochrome-conjugated anti-TCF1 rabbit mAb (C63D9) was purchased from Cell Signal Technology, and fluorochrome-conjugated anti-TOX mAb (REA473) was purchased from Miltenyi Biotec. Intracellular staining of IRF4 uses both IRF4 antibody (D9P5H, Rabbit mAb, Cell Signal Technology) and goat anti-rabbit IgG (H+L) secondary antibody (Thermo Fisher Scientific). T cells underwent fixation and permeabilization and were subjected to IRF4 antibody staining, followed by incubation with secondary antibody. Prior to cytokine staining, cells were stimulated with ionomycin (Sigma-Aldrich), PMA (Sigma-Aldrich), and GolgiPlug (BD Biosciences) for 4 h in complete RPMI 1640 medium at 37 °C. Antibodies targeting murine IFN-γ (XMG1.2) and tumor necrosis factor (TNF)-α (MP6-XT22) were obtained from Thermo Fisher Scientific. Antibodies against murine GranzymB (QA16A02) and Perforin (S16009A) were purchased from BioLegend.

BD LSR II or BD LSRFortessa Flow Cytometer at the Flow Cytometry Core within the Houston Methodist Research Institute was used to assess TILs and splenocytes labeled with various antibodies. FlowJo software (Tree Star) version10 was used to analyze data.

### Statistical analysis

Mice were allocated randomly to either control or treatment groups. Results were expressed as mean ± SD and analyzed with Prism version 8.0.0 (GraphPad Software). The statistical significance of animal survival was evaluated using a log-rank test to determine the *P* values. Tumor growth curves were compared between indicated groups using a 2-way ANOVA (mixed-effects model) or a repeated measures 2-way ANOVA with the Geisser–Greenhouse correction. The *P* values of other measurements were evaluated by using unpaired, 2-tailed Student’s *t* test. Statistical significance was indicated for differences where *P* < 0.05.
