# Supplementary material for: Continuous Expression of Interferon Regulatory Factor 4 Sustains CD8+ T Cell Immunity against Tumor
Source: Research (Wash D C). 2023 Nov 17;6:0271. doi: 10.34133/research.0271 (PMC10765897; doi:10.34133/research.0271)
Supplement: Supplementary 1 — Figs. S1 to S3 [file research.0271.f1.pdf]

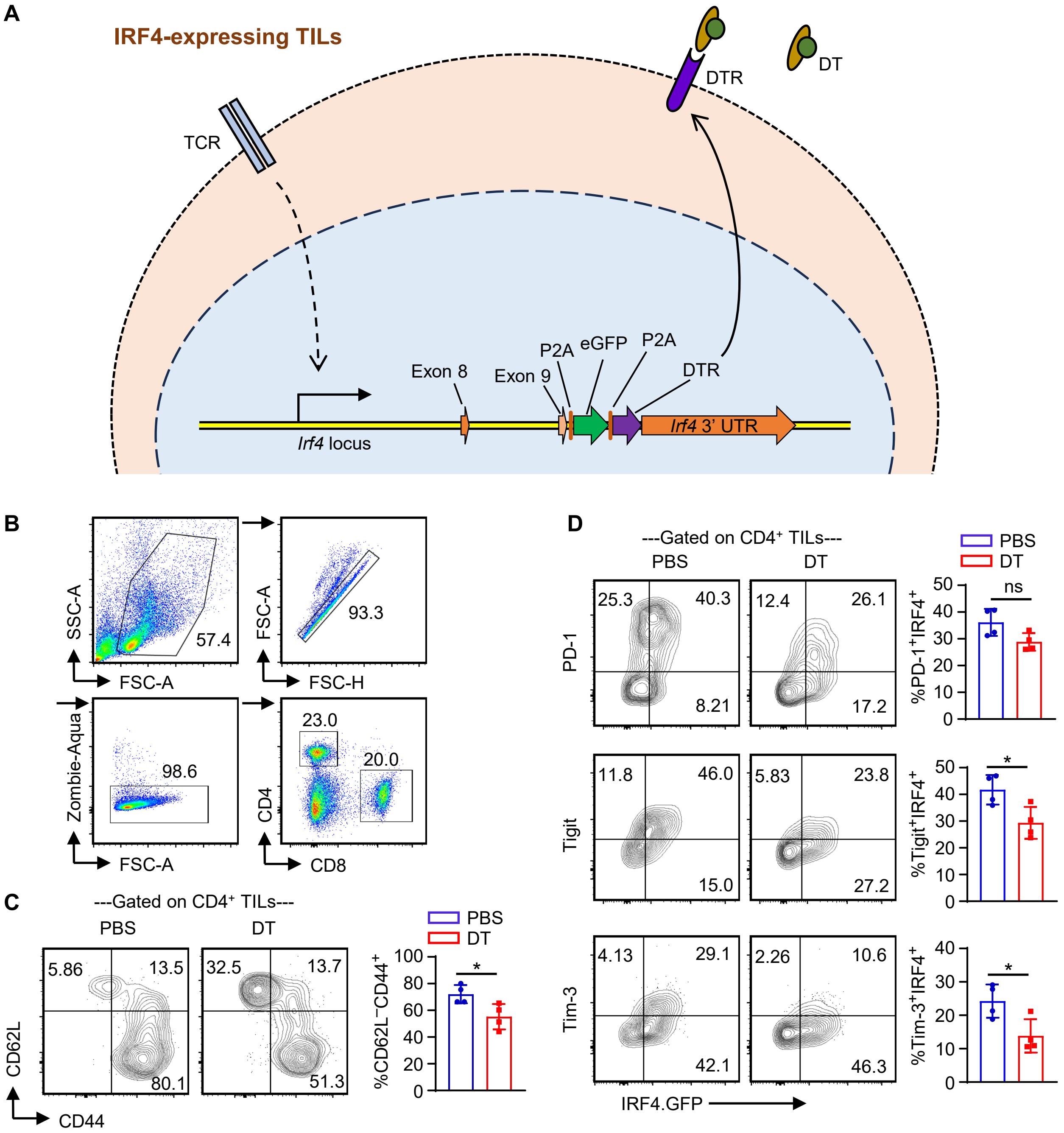

Figure S1. Flow cytometric analysis of CD4^{+} TILs in melanoma, related to Figure 2. (**A**) Diagram illustrating how DT treatment potentially depletes TILs expressing IRF4. For the *Irf4*$^{GFP\text{-}DTR}$ mice, a P2A.eGFP_P2A.DTR_stop cassette was inserted immediately after the last exon of WT B6 mouse *Irf4*. In TILs expressing IRF4, the endogenous *Irf4* expression is associated with DTR expression, rendering these cells susceptible to depletion upon DT administration. (**B–D**) *Irf4*$^{GFP\text{-}DTR}$ mice were subcutaneously injected with 0.1 x 10^{6} B16F10 melanoma cells and received treatments of 25 μg/kg DT or PBS on days 10, 12 and 14 post tumor implantation. TILs were obtained on day 22 for flow cytometry analysis. (**B**) Gating strategies applied during the analysis. (**C**) Percentage of CD62L^{-}CD44^{+} T cells among CD4^{+} TILs. (**D**) Percentages of PD-1^{+}IRF4^{+}, Tigit^{+}IRF4^{+}, and Tim-3^{+}IRF4^{+} cells among CD4^{+} TILs. The data in bar graphs are presented as mean ± SD (n = 4), and statistical significance was determined using an unpaired two-tailed Student's *t* test. ns $P > 0.05$, $^{*}P < 0.05$.

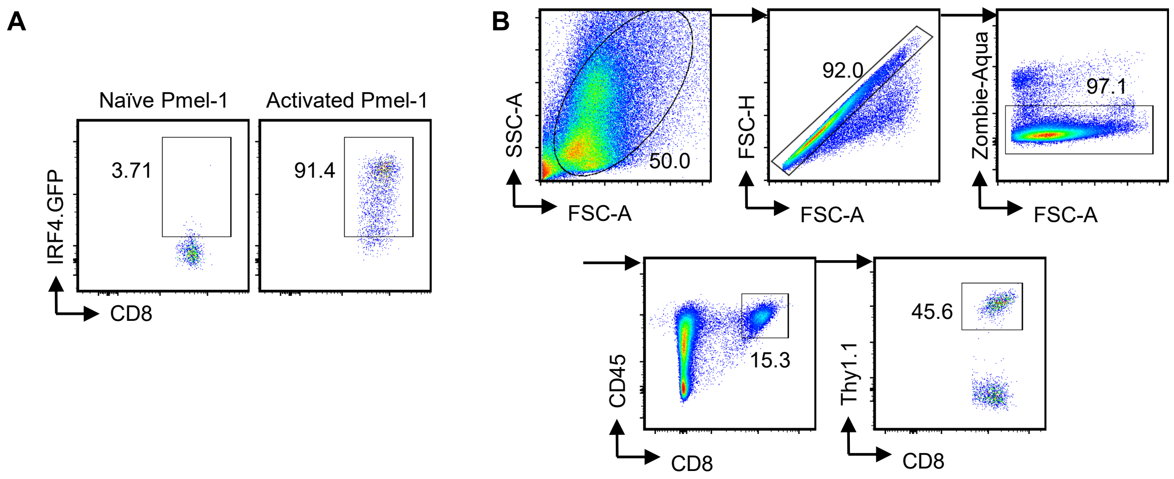

Figure S2. Flow cytometry analysis of Pmel-1 CD8⁺ T cells, related to Figure 3. (**A**) Expression of IRF4.GFP in naïve Pmel-1 cells and *in vitro* activated Pmel-1 cells. (**B**) Gating strategies used to detect the adoptively transferred Pmel-1 cells in the B16F10 melanoma.

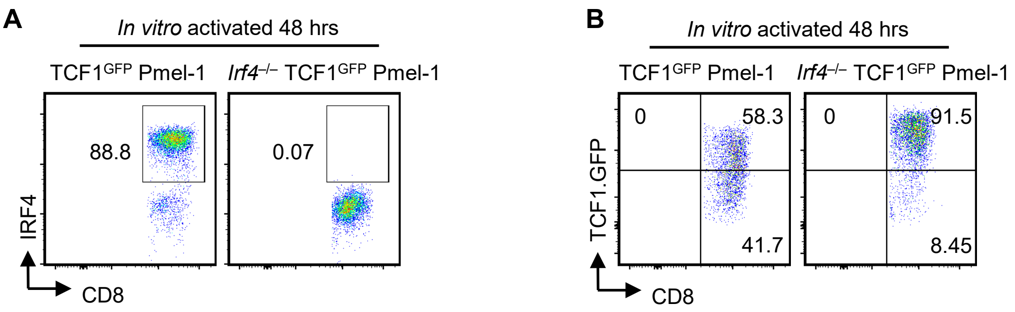

Figure S3. *Irf4*-deficient Pmel-1 cells maintain TCF1 expression following *in vitro* activation, related to Figure 5. Splenocytes from TCF1^{GFP} Pmel-1 or *Irf4*$^{-/-}$ TCF1^{GFP} Pmel-1 mice were *in vitro* stimulated with hgp100$_{25\text{-}33}$ for 48 hrs. (**A**) Representative flow cytometry plots showing the percentage of IRF4^{+} cells among indicated Pmel-1 cell populations. (**B**) Representative flow cytometry plots showing the percentage of TCF1.GFP^{+} cells among indicated Pmel-1 cell populations.
